# Investigating User Proficiency of Motor Imagery for EEG-Based BCI System to Control Simulated Wheelchair

**DOI:** 10.3390/s22249788

**Published:** 2022-12-13

**Authors:** Theerat Saichoo, Poonpong Boonbrahm, Yunyong Punsawad

**Affiliations:** 1 School of Informatics, Walailak University, Nakhon Si Thammarat 80160, Thailand; 2 Informatics Innovative Center of Excellence, Walailak University, Nakhon Si Thammarat 80160, Thailand

**Keywords:** brain–computer interface, brain-controlled wheelchair, electroencephalography, alpha power, motor imagery, EEG neuroheadset

## Abstract

The research on the electroencephalography (EEG)-based brain–computer interface (BCI) is widely utilized for wheelchair control. The ability of the user is one factor of BCI efficiency. Therefore, we focused on BCI tasks and protocols to yield high efficiency from the robust EEG features of individual users. This study proposes a task-based brain activity to gain the power of the alpha band, which included eyes closed for alpha response at the occipital area, attention to an upward arrow for alpha response at the frontal area, and an imagined left/right motor for alpha event-related desynchronization at the left/right motor cortex. An EPOC X neuroheadset was used to acquire the EEG signals. We also proposed user proficiency in motor imagery sessions with limb movement paradigms by recommending motor imagination tasks. Using the proposed system, we verified the feature extraction algorithms and command translation. Twelve volunteers participated in the experiment, and the conventional paradigm of motor imagery was used to compare the efficiencies. With utilized user proficiency in motor imagery, an average accuracy of 83.7% across the left and right commands was achieved. The recommended MI paradigm via user proficiency achieved an approximately 4% higher accuracy than the conventional MI paradigm. Moreover, the real-time control results of a simulated wheelchair revealed a high efficiency based on the time condition. The time results for the same task as the joystick-based control were still approximately three times longer. We suggest that user proficiency be used to recommend an individual MI paradigm for beginners. Furthermore, the proposed BCI system can be used for electric wheelchair control by people with severe disabilities.

## 1. Introduction

Human–computer interaction (HCI) is being researched and developed rapidly. The brain–computer interface (BCI) [1] is an emerging technology that allows direct connections between the brain and a computer. The BCI has been rapidly developed for medical applications, such as rehabilitation, assistive technology, and treatment [2]. The primary users are patients with physical movement disabilities, such as paralysis and spinal cord injury (SCI). Many researchers have developed BCI systems to reduce inequalities and improve the quality of life. The BCI is a beneficial assistive technology used for control and communication by people with severe disabilities [3]. A brain-controlled wheelchair [4] is a popular application for increasing the environmental access for the disabled. An electroencephalogram (EEG) is an electrophysiological signal generated by millions of neurons in the brain. Pulse signal transmission can be used to formulate an electric field through the cerebral cortex, which can be noninvasively measured by placing electrodes on the scalp. A portable EEG device with dry EEG electrodes [5] can yield new research and applications in neurotechnology. Neuroheadsets are key to commercial BCI devices for many applications, such as driver vigilance, education, and entertainment [6,7].

Neurorehabilitation devices using BCI for motor function recovery in stroke patients have been developed into commercial products by employing EEG signals while imagining upper or lower limb movements without actual movement; this is called motor imagery (MI) [8]. Event-related desynchronization/synchronization (ERD/ERS) patterns are usually employed for the left- or right-side motor-imagined detection of the brain regions involved in motor planning and execution [9,10]. Visual evoked potential (VEPs)-based BCI is also a popular and high-efficiency technique for assistive device control via visual stimulation in the transient or steady state to generate P300 [11] and steady-state visual evoked potential (SSVEP) [12], respectively. Moreover, hybrid BCI using a combination of EEG and electrooculogram (EOG) or electromyogram (EMG) signals can be used to build practical assistive devices for smart home applications [13,14,15,16,17]. However, it cannot be used by all people with disabilities, such as ALS patients who have slight loss of muscle function. Therefore, the development of BCI is challenging in terms of communication and control enhancements. Nevertheless, mental and motor imagery have been improved for practical use because the paradigm without stimulation is similar to natural actions. The improvements in the motor imagery-based non-invasive BCI systems can be divided into three methods: (1) a new approach and hybridization of paradigms [3,7,17,18]; (2) classification algorithm designs [19]; and (3) user training systems [20,21]. However, invasive BCI with electrocorticography (ECoG) can yield a higher accuracy than noninvasive BCI. Nevertheless, non-invasive methods can be alternatives in terms of cost and risk.

The research on brain-controlled wheelchairs has attracted attention, and many researchers have proposed techniques and strategies to increase the system and user performances [22,23,24]. Smart wheelchairs with robotic and navigation systems have cooperated with BCI systems for practical systems [25,26]. The ability of a user with different levels of disability or paralysis is a key factor for improving BCI. Some studies have focused on mental tasks and user training protocols to yield high efficiency from the strong EEG features of individual users [27]. Based on previous research, we assumed that the calibration and evaluation of individual users before implementation could be used to improve user performance. Therefore, we propose a technique of command creation for a brain-controlled wheelchair in people with severe paralysis. This study attempted to create an EEG alpha power activity task based on the activated brain areas. The proposed method was divided into two parts: (1) observation of user proficiency of motor imagery and (2) motor imagery-based BCI for simulated wheelchair control. We also aimed to develop a user-friendly BCI system using an EEG neuroheadset for assistive device control. We designed the control creation and translation by utilizing EEG alpha power from three tasks: spatial attention, closed eyes, and left/right motor imagery to control a simulated power wheelchair. We added individual user calibration to seek suitable motor imagery tasks from the limb and hand movements. 

## 2. Materials and Methods

**Figure 1:**
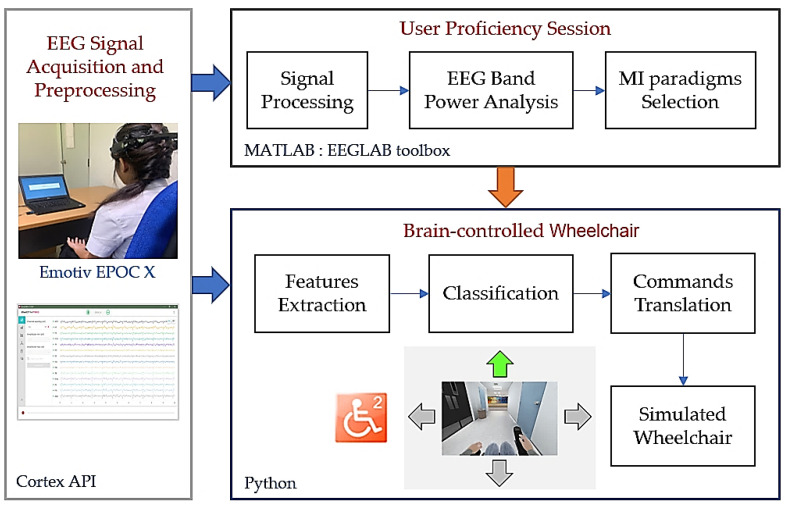
Proposed brain-controlled wheelchair using task-related EEG alpha power from an EEG neuroheadset.

The development of MI-based BCI can be divided into three main parts: (1) optimization of the system by developing algorithms to process and classify brain signals; (2) presentation and development of a paradigm to help differentiate EEG signals; and (3) training to enhance motor imagery of user movements. In this study, we propose a system for exploring the personal paradigm of a user’s motor imagery to obtain a more effective EEG, along with a training plan for medical applications. Previously, there was only a system to train users. The research has increased to include the part examining the efficiency of motor imagery under different positions of the arms and legs in each hemisphere by conducting EEG analysis before performing BCI commands. An overview of the real-time BCI system for simulated wheelchair control with the proposed user proficiency session is shown in Figure 1.

### 2.1. Proposed Paradigms and Commands

In this study, we propose a brain–machine interface system using EEG obtained from an Emotiv EPOC X neuroheadset to generate commands from motor imagery tasks to control the direction of a wheelchair. Four commands for direction control, consisting of going forward, turning left, turning right, and reversing, were created using the proposed command strategies, as shown in Table 1. We used attention to the green arrow to create a forward command. Left and right motor imagery with limb movement paradigms were employed to create the turn left and right commands, respectively. Backward commands were generated by closing the eyes for more than 2 s. In the idle state, the wheelchair stopped. 

**Figure 2:**
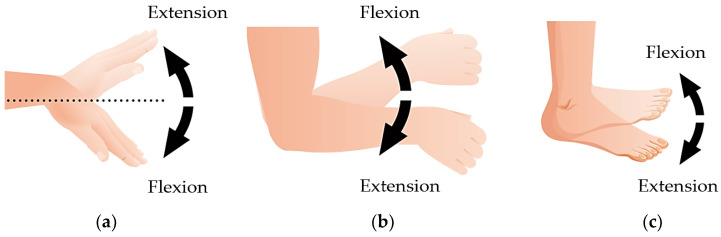
The example of left and right sides of upper and lower limb movement paradigms to investigate user proficiency with motor imagery for paradigms recommendation: (**a**) the left wrist flexion/extension; (**b**) right elbow flexion/extension; (**c**) right ankle flexion/extension.

For investigating the user proficiency with motor imagery, there were left and right sides of the upper and lower limb movement paradigms for the motor imagery. Three joint movements of the upper and lower limbs were used, i.e., wrist flexion/extension, elbow flexion/extension, and ankle flexion/extension. An example of the upper and lower limb movement paradigms is shown in Figure 2.

### 2.2. EEG Acquisition and Preprocessing

**Figure 3:**
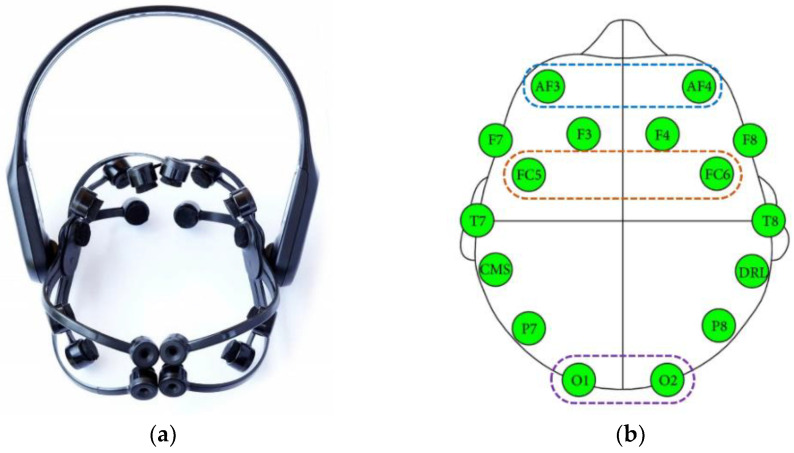
(**a**) 14-channel Emotiv EPOC X; (**b**) electrodes position.

In this study, we used the 14-channel Emotiv EPOC X neuroheadset (shown in Figure 3a), which is a low-cost device which is both flexible and portable. Moreover, the EEG neuroheadset was designed for research on the human brain to acquire professional-grade brain data (https://www.emotiv.com, accessed on 20 September 2022). The electrode positions for EPOC X followed the international 10–20 system (Figure 3b). The 14 electrode positions cover the frontal and prefrontal lobes and the temporal, parietal, and occipital lobes on both sides of the brain at AF3, F3, F7, FC5, T7, P7, O1, O2, P8, T8, FC6, F4, F8, and AF4. The M1 and M2 positions were reference electrodes. P3 and P4 are positioned on the parietal lobe as alternative references for the 14-channel EEG acquisition. Emotiv EPOC X can record EEG signal acquisition at a sampling rate of 256 Hz. During preprocessing, the recorded signals were filtered for power line noise using a 50 Hz notch filter, and a 2 Hz–30 Hz bandpass digital filter was used for motion artifact removal.

Emotiv developed the EmotivPRO software for brain–computer interfaces, neuroscience research, and EEG application development. Moreover, the Cortex application programming interface (API) was designed to stream the acquired data to create third-party applications based on JavaScript object notation (JSON) and WebSocket. Hence, the Cortex API can easily access several programming languages and platforms by EEG data in the JSON format to implement the classification algorithm and commands translation in the Python program for controlling the McGill immersive wheelchair simulator (miWE) [28].

### 2.3. Observations of EEG Alpha Power with Difference Task

Twelve healthy volunteers (six females and six males, aged 22–25) participated in the experiments. To meet the inclusion criteria, all the participants were normal and without disorders. All the participants were informed of the study and read the documentation to participate in the investigation. All the participants signed consent forms (without personal identification), which were kept confidential. All protocols involving human participants were approved by the Office of the Human Research Ethics Committee of Walailak University, which adopted the Ethical Declaration of Helsinki, the Council for International Organizations of Medical Sciences (CIOMS), and the World Health Organization (WHO) guidelines.

**Figure 4:**
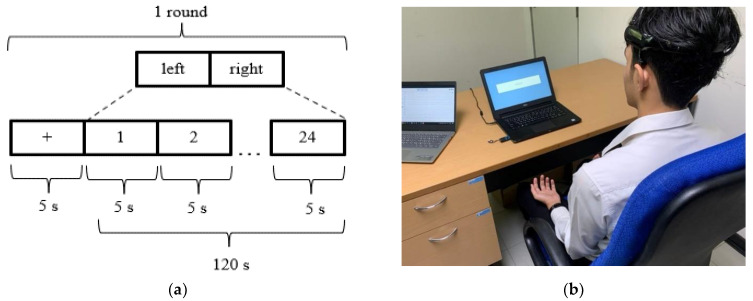
(**a**) Task experiment for motor imagery via upper and lower limb movement paradigms; (**b**) the experimental setup.

The participants performed the experiment using the task sequence illustrated in Figure 4a. The volunteers completed the experiments according to their upper and lower limb movement paradigms. They started by looking at the fixation “+” for 5 s to record the EEG signal in the resting state. Subsequently, each movement paradigm was imagined (Figure 2) for 5 s. The movements were randomly imagined 12 times on both the left and right sides, for a total of 24 times for 125 s per session. Each participant performed ten trials per experiment, twice per movement paradigm. The participants rested for 60 s before moving to the next paradigm. The time the participants spent was approximately 30 min in total. Each subject randomly performed left and right commands, with 20 trials per stimulus pattern and 80 trials per subject. After finishing the motor imagery tasks, each participant collected the EEG during the attention and eye closing stages for 5 s and five times per paradigm.

**Figure 5:**
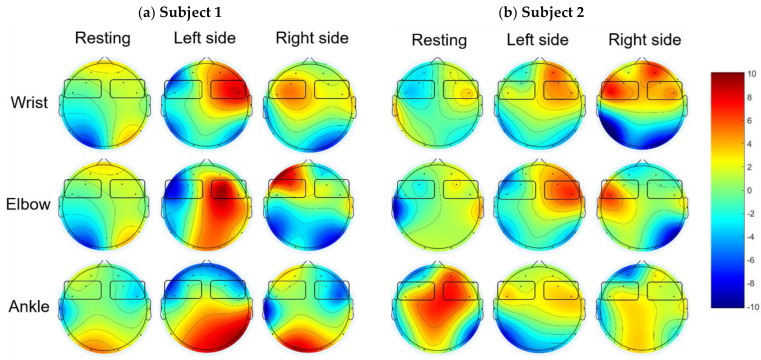
Examples of topographic brain maps of alpha ERD (10–12 Hz) from EEG while subjects 1 and 2 performed motor imagery tasks with left/right side of wrist, elbow, and ankle movements.

**Figure 6:**
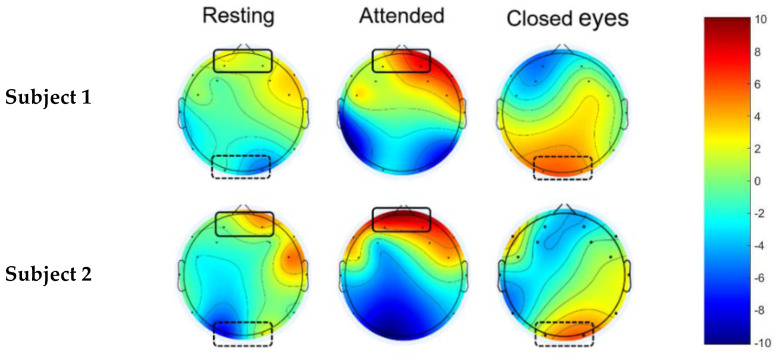
Examples of topographic brain maps of alpha power from EEG while subjects 1 and 2 performed spatial attention and closed eyes.

The recorded EEG signals were filtered using a bandpass FIR filter at 3–30 Hz. The filtered signals were segmented, and topographic brain maps were generated to observe the MI response. All processes were performed using MATLAB (MathWorks) [ver. R2019a], using the EEGLAB toolbox [29]. Based on the grand-averaged brain topographic mapping of the FFT absolute power for all the trials of each subject, we observed the feature pattern for each motor imagery task (Figure 5), spatial attention, and closed eyes (Figure 6). The brain areas of interest were the left–right frontal and occipital areas.

Figure 5 shows examples of topographic brain maps of alpha ERD from EEG while subjects 1 and 2 imagined left/right limb movements. For the left motor imagery, we observed that only the left central (FC5) exhibited a greater response in the alpha band (10–12 Hz), as shown in Figure 5a,b for subjects 1 and 2, respectively. In contrast, the right central (FC6) regions exhibited a greater response from the left central (FC5) region that imagined wrist and elbow movements, as shown in Figure 5a,b for subjects 1 and 2, respectively.

Attention to the green arrow: both sides of the frontal area (AF3 and AF4) exhibited a greater response in the alpha band (8–12 Hz) than in the resting state, according to topographic brain maps of subjects 1 and 2, as shown in Figure 6. For eye closing, the left and right occipital regions (O1 and O2) exhibited a greater response in the alpha band (8–12 Hz) than in the resting state, according to examples of the topographic brain maps of subjects 1 and 2, as shown in Figure 6.

Figure 5 shows that each participant can exhibit a strong ERD response with different movement paradigms. Therefore, we tried to observe user proficiency for motor imagery with various limb movements for the recommended MI paradigms of each user, as shown in Table 2. Our aim was that the recommended MI paradigms could enhance the MI-based BCI. 

## 3. Proposed BCI for Simulated Wheelchair Control

According to the observations of the EEG alpha power in Section 2.3, the EEG signals from channels AF3, AF4, FC5, FC6, O1, and O2 show strong features for attention, thinking, and eye closing. The EEG signals from channels AF3 and AF4 reveal a prominent feature for paying attention to the green arrow. The EEGs from FC5 and FC6 detected alpha ERD while the user imagined upper and lower limb movements (Figure 3). The stop command can be created by closing the eyes to modulate the alpha EEG signals at O1 and O2. The user can generate a forward command by paying attention to the green arrow and a stop command and reverse command by closing both eyes. The turn left command can be generated by imagining moving the left limbs and the turn right command by imagining moving the right limbs. The results in Section 2.3 recommend paradigms from user proficiency for the motor imagery for left and right direction control, as shown in Table 2. The EEG signals were used for real-time processing to detect actions (Table 1) every 2 s to create commands for virtual wheelchair direction control. Conventional EEG alpha features using Welch’s periodogram method and simple classification algorithms for action detection were used for fast computations [30]. The processes of the feature extraction and classification are as follows. 

(1) Calibration: Before using the proposed system, baseline parameters were collected while the user was relaxing for the first 5 s.

The threshold parameter ($T_{e}$) was defined as the baseline relative alpha power in the EEG channels (*e*), that is, AF3, AF4, FC5, FC6, O1, and O2, which were calculated using Equations (1) and (2):

(1) $$BR_{e}\left(\alpha \right)=\frac{P_{e}\left(\alpha \right)}{\left(P_{e}\left(\theta \right)+P_{e}\left(\alpha \right)+P_{e}\left(\beta \right)\right)}$$

(2) $$T_{e}\left(\alpha \right)=1.25*R_{e}{\left(\alpha \right)}_{\;}$$

where $P_{e}$ is the absolute power of the PSDs of the EEG channels (*e*) from AF3, AF4, FC5, FC6, O1, and O2. We used three EEG bands: the theta band (*θ*) (4–7 Hz), alpha band (*α*) (8–12 Hz), and beta band (*β*) (13–30 Hz), without the delta band (1–3 Hz), to avoid motion artifacts. Based on our assumptions, the alpha band should be increased. Hence, the index was defined to allow the difference level to be greater than 0.25 and to multiply $R_{e}\left(\alpha \right)$ by 1.25 as the threshold for the alpha ERD detection.

(2) Feature Extraction: Of the EEG features acquired during stimulation, $R_{e}$ is the relative power spectral density (PSD) of the alpha band of the EEG signals from all the EEG channels, which are calculated using Equation (3), as follows:

(3) $$R_{e}\left(\alpha \right)=\frac{P_{e}\left(\alpha \right)}{\left(P_{e}\left(\theta \right)+P_{e}\left(\alpha \right)+P_{e}\left(\beta \right)\right)}$$

where $P_{e}\left(\theta \right)$, $P_{e}\left(\alpha \right)$, and $P_{e}\left(\beta \right)\;$ are the magnitudes of the PSDs of the real-time acquired EEGs at e = AF3, AF4, FC5, FC6, O1, and O2, respectively.

The alpha ERD response ($D_{e}$), which is the difference of the $R_{e}\left(\alpha \right)$ and $T_{e}\left(\alpha \right)$ values ($R_{e}\left(\alpha \right)\;$ − $T_{e}\left(\alpha \right)$) at *e* = AF3, AF4, FC5, FC6, O1, and O2, can be calculated with Equation (4), as follows:

(4) $$D_{e}=\left\{\begin{array}{c} R_{e}\left(\alpha \right)-T_{e}\left(\alpha \right),\;\;\;\;R_{e}\left(\alpha \right)-T_{e}\left(\alpha \right)>0\\ \;\;\;\;\;\;0\;\;\;\;\;\;\;\;\;\;\;\;\;\;\;\;\;,\;\;\;\;\;\;R_{e}\left(\alpha \right)-T_{e}\left(\alpha \right)<0 \end{array}\right.$$

The output of the process can be obtained as the index of the maximum of $D_{e}$ that is returned from the argument max function (argmax) referring to the largest output, which can be calculated with Equation (5), as follows:

(5) $$i=argmax\left\{D_{AF3}\right.,\;D_{AF4},\;D_{FC5},\;D_{FC6}D_{O1},\;\left.D_{O2}\right\}$$

**Figure 7:**
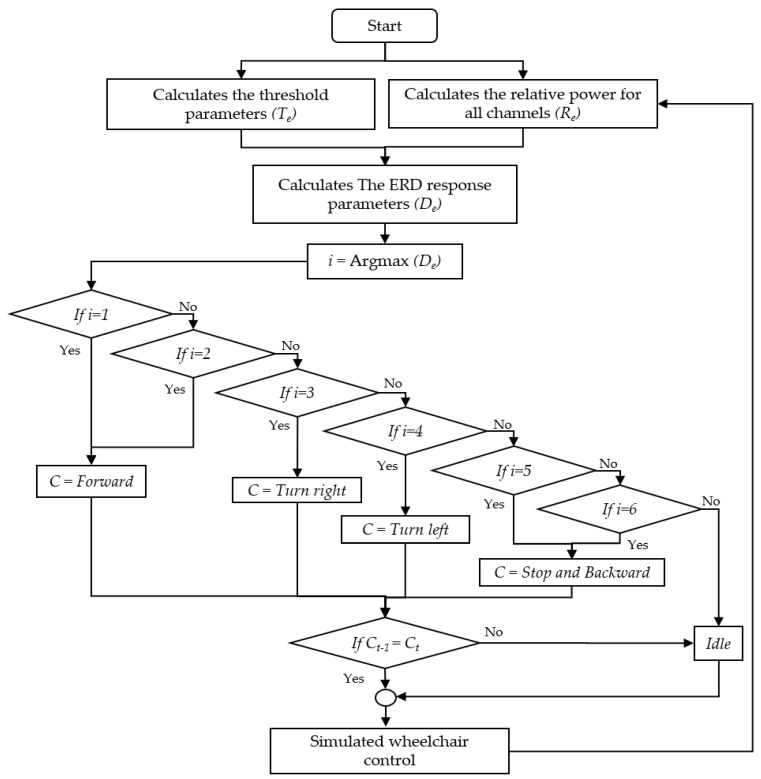
Flowchart of the proposed classification decisions.

(3) Decision Making: We used a simple decision rule to compare the Output values. The four-class classification decision (Figure 7) was generated according to:

if i = 1 or i = 2,    *C* = “Forward”
if i = 3,      *C* = “Turn right”
if i = 4,      *C* = “Turn left”
if i = 5 or i = 6,   *C* = “Stop and Backward”
  Otherwise,      *C* = “Idle”

We also employed a double-check method for command creation by comparing the previous (Ct−1) and present commands (Ct) to avoid involuntary commands (true negative). When they are both the same commands, they are generated. The proposed algorithm establishes a command every 1 s, which requires 2 s for the command output.

## 4. Experiments and Results 

### 4.1. Experiment I: Performance of Using Recommended Paradigm

Before testing, each subject completed a training session of 10 min for each paradigm (wrists, elbows, ankles, and the recommended paradigm) (Table 2) and then proceeded with the experiment. Each subject was assigned to create commands for a virtual simulation of the electric wheelchair movement direction (turning left and right), as shown in Table 3. Each subject performed two trials per paradigm (24 commands). The subject started with the wrists, then the elbow, ankles, and recommended movement paradigm. The subjects took a five-minute break before changing to the next paradigm. Accuracy was collected to verify the proposed user proficiency of the MI paradigm. The experimental results in Table 4 show the average accuracy for each subject. The analysis mainly focused on investigating user performance. The accuracy of each motor imagery paradigm was collected and analyzed for studying between the traditional and recommended motor imagery paradigms. The data were calculated as the mean values and their standard deviation and were expressed as mean ± S.D.

According to the results in Table 4, the maximum accuracy achieved using the elbow and the recommended motor imagery paradigms (Table 2) was 91.7%. The average accuracy of the motor imagery paradigms using wrist movements was 75.3%; using elbow movements was 80.6%; using ankle movements was 75.0%; and using the recommended paradigm was 83.7%. The recommended motor imagery paradigms can yield the highest accuracy rate. The performance of the EEG neuroheadset for an MI-based BCI system was similar to that of the previous MI–BCI systems [31]. The proposed MI–BCI could produce a higher accuracy than the previous studies that used the same EEG devices [32]. Therefore, we employed the subject’s proficiency in motor imagery as a recommended paradigm for the turning left and right commands in a real-time system.

### 4.2. Experiment II: Performance of the Proposed BCI System for Simulated Wheelchair Control

**Figure 8:**
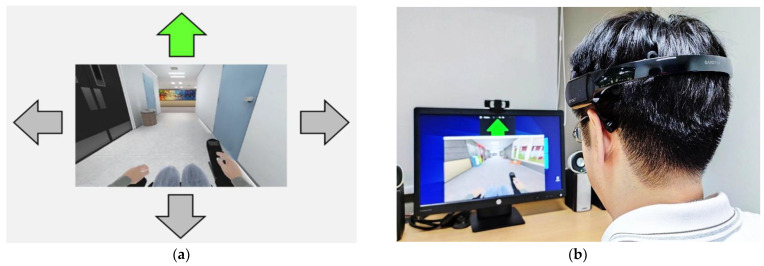
(**a**) Graphic user interface with simulated power wheelchair; (**b**) the example scenario of the simulated wheelchair control during the experiment.

The same group of participants participated in experiment II. Normally, a user’s proficiency level affects the results. Before starting the experiment, we tried to control the participant’s confidence by achieving a greater than 80% accuracy for each command and giving 20 min for a training session. We also recorded the time taken by each subject to steer the simulated power wheelchair using a joystick for user and system evaluations. Each participant was tested using three modalities to independently control the simulated wheelchair, as shown in Figure 8a. The subject generated commands automatically by the proposed algorithms and recorded the time spent steering the simulated wheelchairs to complete routes 1 and 2 using our proposed face-machine interface (FMI) [33]. Each subject performed two rounds for each route. The subject took a ten-minute break before proceeding to the next round. The scenario during the experiment is illustrated in Figure 8b. The time spent from start to stop was recorded to evaluate the proposed BCI system and recommend the motor imagery paradigms. The average time required for each route from two rounds was used to compare with the joystick and FMI control. The data were processed as the mean values and their standard deviation and were expressed as mean ± S.D.

For route 1, the average time required by the joystick control was 54.4 s. The least amount of time obtained using the joystick control was 45 s. The average time required by the proposed MI-based BCI using the recommended paradigm was 211.92 s. The shortest amount of time obtained was 158 s from round 2. For route 2, the average time required by the joystick control was 57.25 s. The least amount of time obtained using the joystick control was 47 s. The average time required by the proposed MI-based BCI using the recommended paradigm was 218.08 s. The shortest amount of time obtained was 168 s from round 1.

After comparing the joystick and BCI controls, we found that the proposed BCI had a lower efficiency than the joystick. The difference between the average times taken by the proposed BCI modality and the joystick on route 1 was 157.54 s and that on route 2 was 160.83 s. All the subjects without BCI experience had difficulty performing and required more training time. Efficiency comparisons with previous works in real-time continuous control [34] showed that the proposed BCI system could produce an elapsed time and command transfer rate similar to those of previous works.

**Figure 9:**
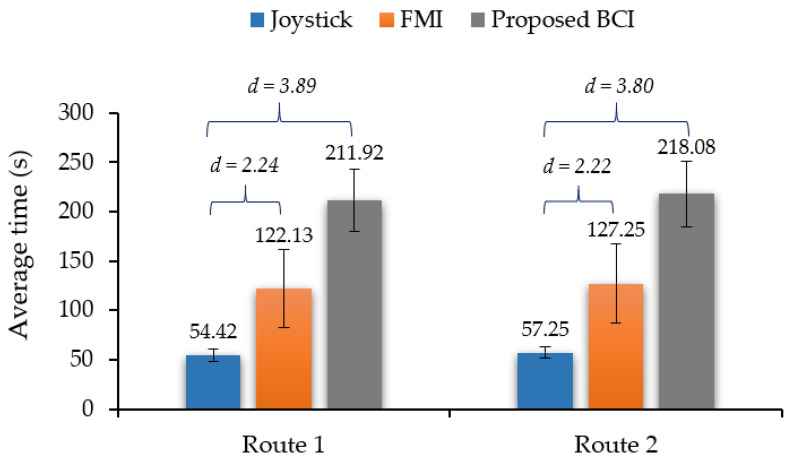
Comparison of average times to complete all routes using the proposed and traditional systems (*d* = time difference ratio).

In Figure 9, using the proposed algorithm, the average time required for individual commands ranged from 173 to 278 s on route 1 and from 173 to 279 s on route 2. Compared with the FMI method, the performance of using the EEG artifacts from the EEG neuroheadsets via jaw chewing and eye winking to control the simulated wheelchair [33] was in the range similar to that from the previous studies. As the features of the EEG signal of the FMI methods are different, this proposed MI-based BCI system required a time of around 90 s on both routes 1 and 2, which is more than that using the FMI method. Moreover, the time difference between the proposed BCI and joystick-based control was approximately threefold.

## 5. Discussion

According to the results in Table 2, most of the subjects could yield a strong ERD using the elbow movement paradigm. From the results of experiment I, using the proposed algorithm, the average classification accuracy of the proposed system for individual commands ranged from 62.5% to 91.7%; maximum average accuracy was achieved by the recommended paradigm; the average accuracy of the recommended paradigm was 83.7%; the average accuracy of the wrist paradigm was 75.3%; the average accuracy of the elbow paradigm was 80.6%; and the average accuracy was 75.0% for the ankle paradigm. We also found that the recommended limb paradigm using the MI proficiency screening for beginners could be used for user training and progression. Therefore, we employed the recommended paradigm for the MI-based BCI for wheelchair control.

According to the results in Table 5, the proposed BCI system for simulated wheelchair control using left/right motor imagery, attention, and eye closing paradigms translated to the turn left, turn right, forward, and backward commands, respectively. All the subjects produced more than 80% accuracy for each command before testing. The results reported the time taken by all subjects to complete the routes. The time required for route 1 ranged from 158 to 280 s, and the time required for route 2 ranged from 168 to 280 s. The average time taken using the proposed system was 211.92 s and 218.08 s for routes 1 and 2, respectively. Compared to the joystick, the difference between the average times taken by the proposed BCI and the joystick on route 1 was 157.5 s and that on route 2 was 160.6 s. Compared to FMI, the difference between the average times taken by the proposed BCI and the joystick on route 1 was 89.8 s and that on route 2 was 90.8 s, as shown in Figure 9. Subjects 2, 5, and 10 with BCI experience demonstrated high efficiency when using the proposed BCI and achieved an efficiency close to our proposed FMI system [33].

However, some suggestions and limitations of real-time motor imagery-based BCI systems using EEG headsets when controlling wheelchair should be reported.

(1) For EEG headset installation, it is necessary to check whether the electrodes are located in the right area and have low impedance during use.

(2) The system still required training sessions for some participants who had trouble with motor imagination in producing apparent features in a proposed BCI system.

(3) To avoid a significant mistake rate over an incredible duration, the system required an auto-calibration system and a monitoring of user fatigue.

(4) For multi-command BCIs, the proposed system yielded lower efficiency than the use of EEG artifacts from the neuroheadset.

## 6. Conclusions

In this study, we proposed the user proficiency of motor imagery via limb movement paradigms for an EEG-based BCI system using the Emotiv EPOC X neuroheadset for the control application. To investigate users’ motor imagery proficiency using brain topographic maps for the limb paradigm recommendations, we verified the proposed MI paradigm using a real-time MI-based BCI for the left/right commands. The recommended paradigm can yield a higher efficiency than the conventional paradigm for the same limb for the left and right paradigms. The proposed user proficiency of the motor imagery method can be used in BCI systems. Moreover, we proposed a BCI system to control a simulated wheelchair by employing the recommended motor imagery, attention, and eye closing actions. The time results for the same task as a joystick-based control are still approximately three times longer. We conclude that user proficiency can be used to recommend an individual MI paradigm to beginners. The studies with healthy people can be applied to investigate user proficiency in motor imagery and to design a training program for patients using BCI-based assistive and rehabilitation systems. Furthermore, the proposed BCI system can be employed for electric wheelchairs or electric devices. For future work, we will implement and verify the proposed BCI system with real electric wheelchairs for practical use by people with severe physical disabilities.

## Figures and Tables

**Table 1:** Proposed action for mental tasks mapping with output commands.

Commands	Actions	Output Commands
1	Attention to the arrow	Forward
2	Imagined left limb movement	Turn Left
3	Imagined right limb movement	Turn Right
4	Closed eyes	Stop and Backward

**Table 2:** Recommended paradigms from user proficiency for motor imagery.

Subjects	Recommended Limb Movement Paradigm	
	Left	Right
1	Elbow	Wrist
2	Elbow	Elbow
3	Elbow	Ankle
4	Elbow	Elbow
5	Wrist	Elbow
6	Elbow	Elbow
7	Elbow	Elbow
8	Elbow	Elbow
9	Ankle	Elbow
10	Wrist	Wrist
11	Wrist	Ankle
12	Elbow	Elbow

**Table 3:** The experimental task of the real-time MI-based BCI.

Sequence No.	1	2	3	4	5	6	7	8	9	10	11	12
Commands	Left	Right	Right	Left	Left	Right	Right	Left	Left	Right	Right	Left

**Table 4:** Results of using various left/right motor imagery paradigms.

Subjects	Average Classification Accuracy (%)			
	Left and Right Sides of Motor Imagery Paradigms			
	Wrists	Elbows	Ankles	Recommended (Table 2)
1	83.3	83.3	75.0	87.5
2	79.2	87.5	70.8	87.5
3	70.8	75.0	79.2	83.3
4	75.0	79.2	83.3	83.3
5	70.8	91.7	75.0	91.7
6	87.5	83.3	83.3	87.5
7	62.5	75.0	70.8	79.2
8	83.3	75.0	62.5	79.2
9	75.0	79.2	75.0	83.3
10	75.0	75.0	75.0	79.2
11	75.0	83.3	79.2	83.3
12	66.7	79.2	70.8	79.2
Mean ± S.D.	75.3 ± 7.20	80.6 ± 5.43	75.0 ± 5.89	83.7 ± 4.14

**Table 5:** The average times taken by all subjects to complete routes 1 and 2.

Subjects	Time (s)							
	Route 1				Route 2			
	Joystick	Round 1	Round 2	Avg.	Joystick	Round 1	Round2	Avg.
1	53	182	192	193	54	204	244	218
2	45	196	176	184	47	172	182	179
3	57	222	242	203	65	184	224	233
4	65	202	188	200	58	198	208	198
5	47	178	184	173	52	168	182	183
6	50	190	212	200	59	210	222	217
7	55	240	260	243	61	246	266	263
8	58	228	208	213	63	198	216	212
9	62	280	272	273	66	266	286	279
10	50	168	158	179	49	190	188	173
11	51	214	200	225	55	236	232	216
12	60	266	242	257	58	248	250	246
Mean ± S.D.	54.40 ± 6.16	213.83 ± 35.00	211.17 ± 35.46	211.92 ± 31.62	57.25 ± 6.06	210.00 ± 31.85	225.00 ± 32.87	218.08 ± 32.98

## Data Availability

The data presented in this study are available upon request from the corresponding author.
